# Contributions to the History, Diagnosis, and Management of Croup

**Published:** 1853-01

**Authors:** John Ware

**Affiliations:** Professor of the Theory and Practice of Medicine in Harvard University


Art. IV.—*Contributions to the History, Diagnosis, and Management of Croup.* By John Ware, M. D., Professor of the Theory and Practice of Medicine in Harvard University.

The following papers on the subject of croup, were originally communicated to the Boston Society for Medical Improvement, and to the Suffolk district Medical Society. The first paper was published several years ago iu the New England Medical and Surgical Journal, and it was subsequently reprinted in connection with the other communication in the Boston Medical and Surgical Journal. Circumstances having led to a reperusal of these papers recently, we have been so deeply impressed with the value of the facts therein embodied, and with the important practical bearing of the considerations deduced by the author, that we have determined to reproduce them, for the benefit of our readers, in the pages of this Journal.

The first paper, it will be observed, gives the fruits of the analysis of *one hundred and thirty-one* cases of croup. This paper was prepared ten years since, but the vast superiority of the method of studying diseases by means of the analytical investigation of recorded cases is evidenced by the fact that results thus elicited do not deteriorate with the lapse of time. Their value is permanent. A work like that of Louis on Typhoid Fever, for example, is not for one generation only. It has a perennial existence, and will preserve for ages to come its original freshness. We look upon this paper of Dr. Ware as one of the most valuable of the contributions to modern science, and its value is not a whit lessened in consequence of the observations, and the philosophical analysis having been made years ago. The results developed in that paper are of great practical importance. The peculiar character which belongs intrinsically to the true, or membranous form of croup; the inconvertibility of other varieties into this form, and the existence of the special character of the latter from the access of the disease, are pathological deductions, the bearing of which on practice is made manifest in the subsequent papers. Pursuing the logical tendency of these deductions, Dr. Ware was led to a plan of treating membranous croup essentially different from that ordinarily adopted. Is this plan an improvement? Dr. W. submits a few observations in answer to this inquiry. A greater accumulation of facts is desirable. If the measures hitherto very generally employed in managing this disease are not appropriate, they must be pernicious. They are of a character too decided to be innocent if not useful. They cannot be neutral, but are potent either for good or evil. The question then to be decided by future observations is one upon which, it may be, many lives are dependent; and it is consequently a point of no small consequence to determine whether the old method should be adhered to or abandoned.

The relative fatality of membranous, and the other varieties of disease generally embraced under the name of croup, in the collection of cases analyzed by Dr. Ware, will not fail to arrest the attention of the reader. And the practical relations of this point are scarcely less important than those pertaining to the treatment of true croup.

If the presence of the fibrinous exudation is the only dangerous, as it is the characteristic element of true croup; and if cases of what are distinguished by the author as spasmodic, catarrhal and inflammatory croup do not in their progress assume this element, consequently involving little or no danger, the impropriety and danger of the indiscriminate employment of the vigorous measures usually resorted to, are sufficiently apparent. The reflecting reader will ask, is not the employment of depletion, antimony, etc., productive of most serious consequences in many instances in which from the pathological character, and the natural tendency of the disease, active interference is not called for?

With reference to the point just referred to, the importance of diagnostic criteria by which membranous may be discriminated from other forms of disease, is sufficiently obvious. And here the observations submitted by the author are of great practical interest. The frequent coexistence of a fibrinous exudation in the pharynx with membranous croup, and the uniform absence of this complication in the other varieties, have not, so far as we know, been disproved by observations made since the publication of the first paper by Dr. Ware.

Regarding, thus Dr. Ware's contributions to the pathology and natural history of croup as among the most important that have been made within late years to practical medicine, and reflecting honor on American medical literature, it is to be regretted that so little is known of them as appears to be the case. This is probably, in a great measure, owing to the fact that not much pains have been taken to bring the papers to the notice of the profession. They have never, we believe, been published except in a few medical Journals which, of course, have not a very general circulation. It is a mortifying fact, and one hardly excusable, that in a late *American* work on the

Practice of Medicine * no allusion is made to Dr. Ware in connection with the subject of croup—a subject with the literature of which his name ought, and will in all future time, be identified. It is with the desire to aid in making our brethren better acquainted with the following contributions that we have gathered them for publication in this Journal, feeling well assured that they will prove in a high degree acceptable to our readers.—*Eds. W. Jour. Med.*

Every physician who has much practical acquaintance with disease, will have observed that there are great differences of character among the cases to which he finds it convenient, in accordance with the custom of medical men, to give the general name of *croup.* He finds that a certain portion of these cases—and by far the larger portion—yield readily to the means which he employs, and very often to the ordinary domestic remedies of mothers and nurses. He has indeed reason to believe that a considerable number of them would spontaneously subside if left to themselves. On the other hand he finds that there are some cases, fortunately but few in proportion to the whole, which exhibit throughout their course, a character of obstinacy that bids defiance to treatment, and which, with few exceptions, pass on to a fatal termination uninfluenced by any remedies he can employ.

Different views may be taken of the nature of these cases. It is believed by some, that the former are not, for the most part, essentially different from the latter; that the difference is more in degree than in kind, or that the difference in the severity and result depends on difference of management, that the favorable character and course of the former, are merely owing to early and judicious treatment, and the fatal event of the latter to the inefficient

* Wood's Practice.

or too tardy application of remedies. A long and I trust a faithful examination of this disease has, however satisfied me that this opinion is not correct. I have been led to believe that there is an original and essential difference in these cases; that those of the first kind are pathologically different from the second; that the former, even if they terminate fatally, which happens in some rare instances, do not terminate in the same way, or at least do not exhibit the same morbid conditions; and that no variety or deficiency of treatment will cause a case of the one kind to assume the character of the other.

I do not, however, mean to imply, that all the cases to which I refer, are capable of being classed under two varieties. Among those which I have characterized as the more mild and tractable sort, we still find great differences in the mode of attack, course, and mode of termination, and also in the degree in which they appear to be influenced by remedies. The object of this paper is to endeavor to contribute something towards determining the nature and extent of the distinctions referred to. With this view I have made an examination of all the cases of croup of every kind which have occurred during the last twelve and a half years, in my own practice, and of this examination I now submit the results. Upon certain points relating to the severer form of the disease, I have included the examination of a number of other cases, extending over a period of twenty five years, witnessed partly in my own practice, partly at dissections, and partly in consultations.

It should be first observed, that, in noting cases in order to this inquiry, I have set down as croup, all those which in the common language of the profession are included under this name—viz., all those which, at any stage of their progress, present a fair question of diagnosis; all those in which is heard that shrill, sharp, ringing cough, which is regarded as the cough of croup, accompanied by a distinct embarrassment of respiration, however, slight, and by some affection of the voice. It follows of course, that many very slight cases must have been included among those on which these remarks are founded—cases which yielded or subsided almost at once. Yet it is right that these should form part of the materials of our examination. When we are in search of means of diagnosis, our attention should be directed to all those cases which have, at any period of their progress, exhibited symptoms that give rise to a well-grounded suspicion of their character. Although many cases which excite the apprehension of severe croup on their first attack, pass away very readily, and by their result show themselves to have been of very moderate severity; yet, on the other hand, it is to be recollected, that many cases which at last terminate fatally, do not, at their beginning, exhibit symptoms, at all more severe, or excite apprehensions at all more serious, than those which have so readily subsided.

Of the cases to which this inquiry relates, that occurred during the period extending from Jan. 1830, to July, 1842, the number is 131. For the convenience of examination, these may be divided into four classes. I do not intend by this arrangement to express the opinion that they constitute four distinct diseases. I would not even be understood to assert positively, with our present amount of knowledge, that they are not different manifestations of the same disease. The purpose now is to speak of them as groups of cases distinguished by certain differences in their symptoms and course, which may or may not be connected with an essential difference in their nature. These classes may be designated, with a view to their probable character and for the purpose of referring to them more intelligibly, by the terms membranous, inflammatory, spasmodic and catarrhal. Of the whole number there were:—

| | Cases. | Deaths. |
|---|---|---|
| Of Membranous Croup | 22 | 19 |
| Inflammatory " | 18 | 0 |
| Spasmodic " | 35 | 0 |
| Catarrhal " | 56 | 0 |
| | 131 | 19 |

In the first class are included those cases in which there is reason to believe that a false membrane has been actually formed, lining the larynx and trachea.

In the second class, those cases in which the symptoms are for the most part, of the same character as in the first, but in which there is reason to believe that no membrane has been formed. The grounds for the opinion formed of the nature of these two classes will be stated subsequently.

The terms applied to the third and fourth classes, require no particular explanation.

The symptoms on which we depend for the diagnosis of croup, relate to the cough, the voice and the respiration.

In the early stage of the first form of croup, the cough is by no means peculiar. In the advanced, it assumes a somewhat different character. In the early period it is sharp, shrill, ringing; it does not vary from that which we hear in the other forms, except perhaps that in some of the less formidable cases it is much louder and more violent at the beginning, than it is in those which prove ultimately more alarming. In the latter period it becomes less loud and ringing, but it is equally sharp—it often becomes almost inaudible, bearing the same relation to a common cough, that a whisper does to the common voice. The cough, then, affords no certain means of distinguishing this form of croup at that period of it in which the diagnosis would be most valuable.

Of the state of the voice nearly the same remark may be made. In the advanced stage of a case it is sufficiently characteristic. It becomes a sharp and almost inaudible whisper. But early in the disease it is not always affected at all; and if it be, cannot with certainty be distinguished from the hoarse voice of common catarrh.

The condition of the respiration affords us far more important information. In the early period of the disease, however, when we most need means of diagnosis, it is not a symptom which always attracts attention, even from the physician; much less from others who are around the patient. The common description of the breathing in croup, does not apply well to the beginning of the membranous variety. It seems rather taken from cases of a less dangerous kind, in which the breathing is from the first, loud, harsh, suffocative; attended with great efforts, and much loud coughing; creating great alarm, and calling at once for efficient means of relief. But the breathing in membranous croup does not excite attention in the very commencement of the disease. It is comparatively quiet and unobtrusive. Its true character is not at once to be detected, but only by a careful and accurate observation. The patient has not the ordinary aspect of difficult breathing; in fact, the breathing is not difficult at the very first. He probably experiences no distress. There is no real deficiency in the performance of the function, and no obvious embarrassment. There is only a little more effort in drawingin the air, and a little more force exercised in its expulsion whilst the amount of air admitted and expelled is fully equal to the necessities of life. This perhaps would not be noticed on a casual glance at the patient, but will be at once perceived on attending to the muscular movements subservient to the functions, which are—to use an expressive French term—somewhat exalted. It is indicated very soon, also, by a slight dilatation of the nostrils, and a little whiz or buzz accompanying the passage of air through the rima glottidis. This sound is distinguished either by placing the ear near the mouth of the patient, or by applying the stethescope on the back of the neck, or directly upon the upper part of the larynx.

This at its very beginning is the essential respiration of membranous croup, and it affords far more aid in diagnosis than either the cough or the voice. It is not, however, always found as pure as has been described. It is often mingled with, and obscured by, other sounds. Thus the disease is often attended by paroxysms of irregular and spasmodic breathing, accompanied by violent muscular efforts and great distress, and of course producing other and more obvious sounds than those described. There is often also present in the air passages, either above or below the glottis, a quantity of mucus, giving rise to a constant or occasional rattling, which seems to mask the proper sound of croup. These adventitious sounds, being also as frequently heard in the other forms of croup, are therefore of no service in diagnosis. Generally there are intervals of relief from these superadded symptoms, especially immediately after vomiting or bleeding, but the essential breathing of the disease will be found to be unchanged and unmitigated in these intervals of ease; although the apparent relief may be so considerable as to give rise to strong, but fallacious hopes of recovery.

We occasionally hear, in cases of considerable enlargement of the tonsils, a kind of breathing which closely resembles the early breathing of croup. Usually in such patients the respiration is loud, sonorous, unequal and irregular, but in a few it is quiet, steady, with a muscular effort occasioned by a mechanical obstruction like that in croup. The distinction between them can, however, be readily made, by attending carefully to the seat of the obstruction, which is above the rima glottidis in the one case, and at it in the other; by the sound of the cough and voice, which are not croupy, and by the fact that the obstruction varies in degree and sometimes vanishes, with change of position.

I have endeavored to describe this respiration as it exists in its slightest appreciable degree, at the earliest period of its manifestation. As the disease advances, it becomes very strongly marked, whilst the condition on which its peculiar character depends, viz., a mechanical narrowing of the orifice through which the air passes, becomes much more obvious.

The muscular effort, in the latter stage, becomes very strong, both in inspiration and expiration. During inspiration, whilst all the muscles concerned in it are in the highest state of activity, the mechanical impediment against which they act, is often strikingly displayed by the falling in of the soft part about the neck and clavicles, at the epigastrium, and between and along the lower edge of the ribs—the air not passing in through the narrowed opening of the glottis so rapidly as the dilation of the chest by the increased muscular effort would render necessary. The expiration is chiefly characterized by the amount of force employed to expel the air. In health the expiration is easy and accompanied by little effort. Where there is no unusual obstruction, the mere tendency to collapse of the lungs would be sufficient for the expulsion of the air, as we see in the dead body; so that the walls of the chest have merely to follow up this contraction, without adding to its force by any muscular effort. But in croup, this is not enough; and we often find that the air is blown out forcibly against the mechanical resistance occasioned by the disease. We find the same strong contraction of the muscles concerned, especially of the abdominal muscles, which is observed when air is blown out forcibly through a narrow passage.

This is the proper breathing of croup; becoming more and more intense as the disease approaches its termination, till the whole life of the individual seems, as it were, to concentrate itself in this one effort. The patient in this extreme condition seeks, by a multitude of changes of place and position, to find some alleviation of his agony; the cough, and with it the voice, have become nearly extinct; and his inarticulate appeals and beseeching looks for relief to those from whom he is accustomed to look for it, constitute one of the most touching scenes which we are called upon to witness in the practice of medicine. Happily the extreme suffering usually, though not always, subsides towards the close of life, and death takes place at last with comparative ease.

In the advanced stage of croup, the breathing is often modified by circumstances other than the mere mechanical obstruction at the upper part of the larynx. After a certain period the false membrane is in some places separated from its adhesion to the mucous surface, by the secretion of pus. The passage of air to and fro, and the efforts of coughing, detach it partially from its adhesion, and break it up more or less into shreds, which however still adhere at one of their ends. These rugged portions of membrane, mingled with the pus, move up and down the air passages, causing some variety in the sounds and also in the actual difficulty of breathing. Death is sometimes very suddenly produced by a collection of this material into a mass which becomes impacted in, and thus plugs up, either the upper or lower part of the larynx. This at least, from the state in which the parts are found on dissection, would appear to be the mode in which death takes places.

The respiration may also be modified in croup from a congestion or inflammation of the lungs, which occasionally supervenes. The embarrassment of respiration has also sometimes appeared to be increased by an accumulation of air in the lungs, which arises from a deficient balance between inspiration and expiration. Owing to the greater ease with which we can make extraordinary and continued effort of inspiration than we can of expiration, a greater quantity is admitted than can be readily expelled, before the suffocative feeling of the patient impels him to a new effort for relief.

But although there may be a combination of the respiration of this disease with that produced by other affections of the throat or lungs, yet the respiration of croup is in its nature and character essentially distinct from them. In them the difficulty of breathing and the unusual muscular effort, may arise from a variety of causes, producing great varieties in the modes of dyspnœa; in croup, the one essential condition is the mechanically contracted state of the passage through which the air passes, and all the peculiarities of the dyspnœa proceed from this condition. In one particular, the breathing of asthma resembles that of croup, viz., in the intensity of the effort by which the current of air is made to move in both directions against a mechanical resistance; but the point of the resistance, and consequently the other circumstances of the function prevent the resemblance from extending to other points.

The *first* form of croup, then, is distinguished by the cough, the voice, and by a peculiarity of the respiration which I have attempted to describe, and which for the sake of distinguishing it in this essay, may be called *intense*.

In the cases of the inflammatory croup, which constitute the *second* form of the disease, the condition of the voice, cough and breathing, are precisely the same as in the cases of the first class. There is no certain way by which, so far as these symptoms are concerned, cases of the one kind are to be distinguished from those of the other. The cases enumerated among the second class, were of all degrees of severity, but not one of them was fatal. Cases, however, of croup which terminated fatally, and in which no membrane was found on dissection, are recorded upon the best authority. To those we shall have occasion to advert hereafter. In addition to the symptoms proceeding from the character of the cough, voice and respiration, I have noted in a few examples of this form of the disease, a tenderness of the larynx on pressure.

As cases of this class are then usually favorable in their termination, whilst those of the first are usually fatal, the diagnosis between them, in the early stages especially, becomes of very great importance, both as regards prognosis and treatment. Of the means by which this distinction may probably be made, and of the grounds for believing these two to be essentially distinct diseases, and not different states or conditions of the same disease, I shall take occasion to speak, after considering the other two classes which have been enumerated.

The *third* includes certain cases which are generally designated as *spasmodic croup*, and sometimes as *spasmodic asthma*. The attack is always sudden, and usually occurs after the subject has been, for some time, asleep. Very often it occurs in the evening, during the first sleep of the child, before its parents have retired to bed; but perhaps as frequently at a later hour of the night, or very early in the morning. The patient wakes in great distress for breath. His inspiration is attended with great effort, it is loud, ringing, shrill, somewhat resembling the hooping inspiration of hooping cough, but louder and more sonorous. The expiration is comparatively quiet and easy. The voice at the same time, is hoarse and broken, and there is a loud, hoarse barking cough which closely resembles that of the preceding kinds, and indeed alone, would not serve as a mark of distinction from them. These cases seem occasionally to arise from indigestion; but more frequently we can trace their occurrence to cold, especially as they have been often preceded for a few days by symptoms of catarrh. When left to themselves they will usually subside spontaneously, but from their suddenness and violence, they cause great alarm, and call for immediate assistance. They rarely fail to yield to an emetic or venesection, leaving behind them for a longer or shorter period, rarely for more than twenty-four hours, some hoarseness and some degree of the croupy sound of the cough, with a little huskiness or stiffness of breathing. At no period is there any proper *intensity* of respiration.

These cases, from their suddenness, the time of the attack, the great violence of the first symptoms, and the consequent alarm which they create, produce a stronger impression on the minds of common observers, and even of many practitioners, than those of the other kinds. This mode of attack is most closely associated in their minds with the term croup; and it is regarded as tending, if not checked, to terminate in the same state of things with cases of the first class. So far as the cases before us are concerned, however, this never happens, and of the whole number included under this examination, no one proved fatal.

The fourth class includes cases not falling under either of the above, and yet frequently presenting a very close resemblance to them. The subjects usually exhibit at first the symptoms of common catarrh. After a few days, the voice becomes hoarse; the cough becomes croupy, and there is tightness, oppression, and some approach to the croupy sound of respiration; there is, however, no intense or exalted action of the respiratory muscles, and no indication of that mechanical impediment to the current of air which exists at the rima glottidis in the two first forms of the disease. Still the resemblance is sometimes quite close enough to cases of the same forms, in their earlier stage, to occasion some anxiety, and there is also sometimes a sudden attack of dyspnœa, with loud, shrill and sonorous breathing, which imitates the symptoms of the third form, and is perhaps to be regarded as an attack of the same kind.

The cases of this form yield gradually, the croupy character wearing off in a few days, and leaving behind simply catarrhal symptoms. I suppose them, from the mode in which they came and go off, to be properly a catarrhal inflammation of the mucous membrane covering the organs of voice. We frequently observe that the catarrhal affection of the same membrane which occurs in the first stage of measles, is accompanied by the same croupy symptoms as those which have been now described—going off with the other catarrhal symptoms. In a few instances the attacks of this form of croup have terminated in severe bronchitis, or in inflammation of the lungs themselves. But among the 56 cases included above, there was no one fatal.

Having thus described these several forms of this disease, and stated in general what seemed to be their nature, the question now arises as to the justice of the distinction which has thus been assumed to exist. Is there any sufficient ground for such a distinction? Are these different cases different diseases? Are not the favorable ones, which constitute so large a proportion of the whole number, similar in their nature to the more severe; but either of less severity in their origin, or else modified and controlled in their course, by the influence of treatment? These questions it is obviously of great importance, to the prognosis and treatment of cases in question, to be able to answer correctly. If we can with regard to a large proportion of them, confidently predict from the outset a a favorable issue, the practitioner and the friends will be saved much unnecessary anxiety, and the patient many annoying and debilitating remedies.

I proceed, therefore, to state the grounds for a belief that the first form of croup is a disease essentially distinct from all others, and that it depends on a peculiar pathological condition to which they have no tendency. Whether there be any equally marked distinction between the other froms it is not of the same practical importance to determine; and as we have no sufficient materials for a satisfactory inquiry into this question, our attention will be confined to the evidence for the distinct character of the first form.

Every physician is familiar with an affection of the throat, both in adults and children, consisting in an inflammation of the mucous membrane, of that peculiar character which produces the effusion of a layer of coagulable lymph, or false membrane. The connection of this affection of the throat with croup, was long since pointed out; and it is well known to practitioners among us, that this complaint known familiarly, though inaccurately, under the name of "ulcerated sore throat," often accompanies or is followed by croup, and that croup thus connected is peculiarly fatal in its character. This circumstance in the history of croup, was many years since strongly impressed upon my mind by an eminent practitioner in this neighborhood.* I was in consequence led, in all cases of croup, subsequently to this period, to make a careful examination of the fauces, with a view of determining exactly the extent to which this visible affection of the throat was connected with the more important disease.

Two causes prevent the completeness of these observations. We are very apt, in making record of cases, especially of those which appear of a slight degree of severity, to omit the *noting* of negative facts, even when they have been actually the objects of attention. Hence, although I have very rarely failed to examine the fauces in any case of supposed croup, I have often in the lighter cases, and sometimes in the severer, failed to note their condition. The second cause of incompleteness is the

* Dr. William J. Walker.

impossibility in some patients, from their terror and consequent resistance, of getting such a view of the parts as would authorize us to pronounce decidedly what their state is. Notwithstanding these circumstances, the state of the throat has been noticed and recorded in a sufficient number of cases to afford very fair materials for inference.

With a view to this examination, I may include a considerable number of other cases, besides those which constitute the particular subjects of inquiry in this paper, which have been noticed at other times, or in the practice of my friends. Including these cases with the 22 above referred to, I have memoranda, more or less complete, of 39 cases of what I have denominated membranous croup. The state of the fauces was observed and noted in 33, and of these, in 32 a false membrane was present; most frequently and sometimes only on the tonsils, sometimes on other parts also, as the palate, uvula and pharynx. In one case, no such membrane was present; but it was found to exist in the larnyx after death. In 3 of these 33 cases, recovery took place; all the others were fatal. In 14, an examination was made after death, and the usual appearances were found to exist in all of them.

On the other hand, I have memoranda of 109 cases of what I have classed as the other forms of croup, and of these the state of the tonsils and fauces was noted in 45. In no one was there such a condition of the parts as was found to exist in the membranous form. In 3 cases there was indeed a thin, slight exudation on the tonsils, of the color and appearance of starch, like that which is sometimes seen on the edge and surface of the tongue. This I apprehend to be a formation of an entirely different nature from that which exists in the other class of cases. Of the 45, 12 were of second, 11 of the third, and 22 of the fourth class.

From this statement, it seems probable that the appearance of a false membrane upon the tonsils or other visible part of the throat, in a case of croup, may be regarded as a pretty certain diagnostic sign that it is the membranous form of the disease; and its absence as a pretty certain indication that it is one of the other forms. Still there will be exceptions. There will be cases in which the membrane is formed in the larnyx, although it has not appeared in the throat; and there may be those in which a membrane exists in the throat, unaccompanied by a similar condition of the air passages. Of the former I have recorded one example; of the latter, none. How frequent such exceptions will be, must be determined by more extensive observation. If they are not more frequent than they have been among the cases here recorded, the observation of this symptom will afford a sufficiently safe guide, since of 75 cases in which it was looked for and the result noticed, it failed as a diagnostic sign in but a single instance.

[To be Continued.]

